# Editorial: Current perspectives on *Colletotrichum*: from molecules to ecosystems

**DOI:** 10.3389/ffunb.2026.1817698

**Published:** 2026-03-24

**Authors:** Riccardo Baroncelli, Lars Matthias Voll, Daniela Elisabeth Nordzieke

**Affiliations:** 1 Department of Agricultural and Food Sciences, University of Bologna, Bologna, Italy; 2 Department of Biology, Molecular Plant Physiology, Marburg University, Marburg, Germany; 3 Department of Genetics and Eukaryotic Microorganisms, University of Göttingen, Göttingen, Germany

**Keywords:** antagonistic fungal interactions, *Colletotrichum*, genome and transcriptome analysis, hemibiotrophic crop pathogens, host signals, indolic phytoalexins

The genus *Colletotrichum* represents one of the most diverse and economically relevant groups of plant-associated fungi (Dean et al., 2012). With nearly 350 species now recognized and grouped into 20 species complexes spanning saprophytic, endophytic, and pathogenic lifestyles, the genus occupies a remarkable breadth of ecological niches (Talhinhas and Baroncelli, 2021, 2023). Among these, the pathogenic members are notorious for causing anthracnose, a disease that damages cereals, vegetables, fruits, and ornamentals worldwide. Some species display astonishingly broad host ranges, while others are highly specialized, infecting only a single plant species (Cannon et al., 2012). At the core of their pathogenic strategy lies a hemibiotrophic lifestyle: an initial biotrophic phase characterized by stealthy colonization, followed by a necrotrophic stage in which fungal growth coincides with extensive host tissue destruction. This complexity not only makes *Colletotrichum* a devastating plant pathogen but also a fascinating model system for studying fungal evolution, adaptation, and plant–pathogen interactions (O'Connell et al., 2012; Baroncelli et al., 2024).

The Research Topic “Current Perspectives on *Colletotrichum*: From Molecules to Ecosystems” was launched to capture this breadth, inviting contributions ranging from detailed molecular analyses to applied approaches and ecosystem-level perspectives. The seven articles assembled here reflect this ambition and, taken together, provide an integrated view of the state of *Colletotrichum* research.

One of the longstanding challenges in the field has been taxonomy. Morphological traits often fail to distinguish closely related species, leading to ambiguities in identification and communication. The article “Can whole genome sequencing resolve taxonomic ambiguities in fungi? The case study of *Colletotrichum* associated with ferns” (Menicucci et al.) shows how genome sequencing is helping to overcome these difficulties by offering a higher resolution for delimiting species boundaries. At the same time, understanding genome structure itself provides insights into adaptation. The article “What lies behind the large genome of *Colletotrichum lindemuthianum*” (Lopes da Silva et al.) illustrates how genome expansion, largely due to repetitive elements and effector diversification, can underpin the ability of pathogens to interact with and overcome host defenses. These studies highlight how genomics has become indispensable for unraveling both evolutionary relationships and pathogenic potential (O'Connell et al., 2012)

While genomes provide the blueprint, morphology offers initial insights into how fungi interact with their environment. The study “Morphological variations and adhesive distribution: a cross-species examination in *Colletotrichum* conidia” (Bedsole et al.) sheds light on conidial form and function. Variation in conidial adhesiveness is not a trivial trait; it determines how successfully spores attach to host surfaces, a critical first step in colonization. These findings emphasize that subtle morphological differences can have significant ecological and pathogenic consequences, linking microscopic features to infection biology. Moving slightly further downstream in the infection process, the article “Conserved perception of host and non-host signals via the a-pheromone receptor Ste3 in *Colletotrichum graminicola*” (Rudolph et al.)sheds light on signaling processes underlying the recognition of host roots by *C. graminicola*. The study demonstrates how conserved G-protein coupled receptors, originally characterized in mating processes, have been co-opted to perceive the activity of host proteins, such as peroxidases and the abundance of secondary metabolites, such as diterpenoids, in the rhizosphere. This elegant retooling of ancient molecular machinery illustrates the evolutionary flexibility of these fungi and highlights how conserved pathways can be adapted for novel functions.

Of course, attempted fungal invasion does not go unchallenged. Plants have evolved layered defense systems, often involving plant secondary metabolites. The study “Response of the Arabidopsis indolic secondary metabolite network to infection with *Colletotrichum higginsianum*” (Seufer et al.) examines the role of indolic glucosinolates, camalexin, and indole-carboxylic acid derivatives in *Arabidopsis* defense against *C. higginsianum* and the response of the indolic metabolite network after *C. higginsianum* challenge. By analyzing higher-order mutants, the authors reveal the presence of a, albeit low-efficiency, metabolic pathway from myrosinase-based cleavage of indolic glucosinolates back into the indole acetonitrile pool. The study further reports that, of all the indolic secondary metabolites, only camalexin showed a detectable effect on *C. higginsianum* colonization. In a complementary systems-level approach, “Dual-RNA-sequencing to elucidate the interactions between sorghum and *Colletotrichum sublineola*” (Vela et al.) captures both sides of the interaction simultaneously, offering an unprecedented view of the dynamic transcriptional changes of the host and pathogen during infection. Such integrative approaches exemplify how multi-omics can disentangle the molecular dialog at the heart of disease.

However, *Colletotrichum* research is not confined to the laboratory or to a single plant–pathogen dyad. A striking ecological perspective is provided by “The yeast *Wickerhamomyces anomalus* acts as a predator of the olive anthracnose-causing fungi” (Amorim-Rodrigues et al.). This study reveals direct antagonistic interactions between microorganisms, highlighting how biotic pressures in the environment can influence pathogen success. Beyond its ecological significance, this finding points to novel avenues for biocontrol, where naturally occurring microbial antagonists could help manage diseases that threaten crop production.

So, what does this Research Topic tell us about the future of *Colletotrichum* research? First, it reinforces the power of genomics for both taxonomic clarity and functional insight. As genome sequencing becomes more accessible, comparative studies will continue to illuminate the evolutionary trajectories of this diverse genus. Second, this Research Topic highlights the importance of integrating multiple levels of analysis, from morphology and signaling pathways to chemical defenses and ecological interactions. Each perspective contributes a piece to the puzzle of how *Colletotrichum* thrives in such varied environments. Finally, this Research Topic reminds us that applied and ecological angles are essential complements to molecular studies. In a world where agriculture faces increasing challenges from climate change, global trade, and fungicide resistance, translating fundamental insights into sustainable disease management remains a priority.

In sum, this Research Topic succeeds in presenting *Colletotrichum* as more than just a plant pathogen. It is a genus that embodies evolutionary innovation, ecological interconnectedness, and applied relevance. The contributions gathered here, ranging from molecules to ecosystems, provide a strong foundation for future research and underscore the need for interdisciplinary approaches to address both scientific and agricultural challenges.
